# Complete Genome Sequence of Anguillid Herpesvirus 1 Isolated from Imported Anguilla rostrata (American Eel) from Canada

**DOI:** 10.1128/mra.00829-22

**Published:** 2022-11-29

**Authors:** Sajal Kole, Hyoung Jun Kim, Sung-Ju Jung

**Affiliations:** a Department of Aqualife Medicine, Chonnam National University, Yeosu, Republic of Korea; b OIE Reference Laboratory for VHS, National Institute of Fisheries Science, Busan, Republic of Korea; Queens College CUNY

## Abstract

We report the full-length genome sequence (compared to reference sequences) of a variant strain of *Anguillid herpesvirus 1* (AngHV-1) isolated from imported Anguilla rostrata (American eel) from Canada. This should help to further identify such viruses in the North America.

## ANNOUNCEMENT

*Anguillid herpesvirus 1* (AngHV-1) is an important eel virus belonging to the genus *Cyprinivirus* of the family *Alloherpesviridae* which poses a serious threat to both farmed and wild eel species worldwide (1–3). AngHV-1 is an enveloped double-stranded DNA virus which causes hemorrhagic skin lesions and destruction of gill filaments (4). AngHV-1 is frequently detected in Anguilla anguilla from the Netherlands (5), Denmark (6), the United Kingdom (7), and Poland (8); in Anguilla japonica from Japan (4) and Taiwan (9); in Anguilla bicolor from South Korea (10); and recently, in Anguilla marmorata from Vietnam (11).

Taking cue from this, we conducted a virus surveillance program for the 2017–2019 period with American eel (Anguilla rostrata) imported (*n* = 5 eels/consignment) from Canada in oxygen-supplied bags directly to the laboratory without being exposed to South Korean water. For this, gill tissues were collected and aliquoted for DNA extraction, followed by PCR screening using a primer targeting the AngHV-1 DNA polymerase gene (5), and for virus isolation in the eel kidney cell line (EK-1). PCR-positive gill homogenates were inoculated using the limiting dilution method (5 times) in EK-1 cells. After development of a complete cytopathic effect involving rounding and enlargement of the cells, the supernatant containing AngHV-1 was collected, confirmed by PCR assay, and propagated in tissue culture flasks. The harvested AngHV-1 with a virus titer of 10^6.8^ 50% tissue culture infective dose (TCID_50_) was subjected to genomic DNA (gDNA) extraction using the high-pure PCR template preparation kit (Roche).

Double-stranded library preparation was performed with 1 mg sheared gDNA using the MGIEasy DNA library prep kit (MGI), followed by quantification using the QuantiFluor ONE double-stranded DNA (dsDNA) system (Promega). The library was circularized, digested, and cleaned by incubating it at 37°C for 60 min. Subsequently, a DNA nanoball (DNB) was prepared by incubating the library at 30°C for 25 min using DNB enzyme, followed by quantification using the QuantiFluor single-stranded DNA (ssDNA) system (Promega). Finally, sequencing of the prepared DNB was conducted using the MGISEQ system (MGI), yielding 29,772,167 reads (150-bp paired-end format) with a fold coverage of 31,543.4×. The raw data were trimmed using Cutadapt ver. 1.9, and a contig sequence was produced using the CLC Genomics Workbench ver. 20.0.4 *de novo* assembler (Qiagen) with default settings. The genome completeness was confirmed by mapping the filtered data into the contig sequence using the Map to Reference tool of Geneious ver. 2021.1.1 software, with default parameters. The detailed annotation of the completed sequence was manually corrected by referencing it against the sequences found under GenBank accession numbers FJ940765 and KX027736 using SnapGene software ver. 5.3.2 (GSL Biotech LLC).

**FIG 1:**
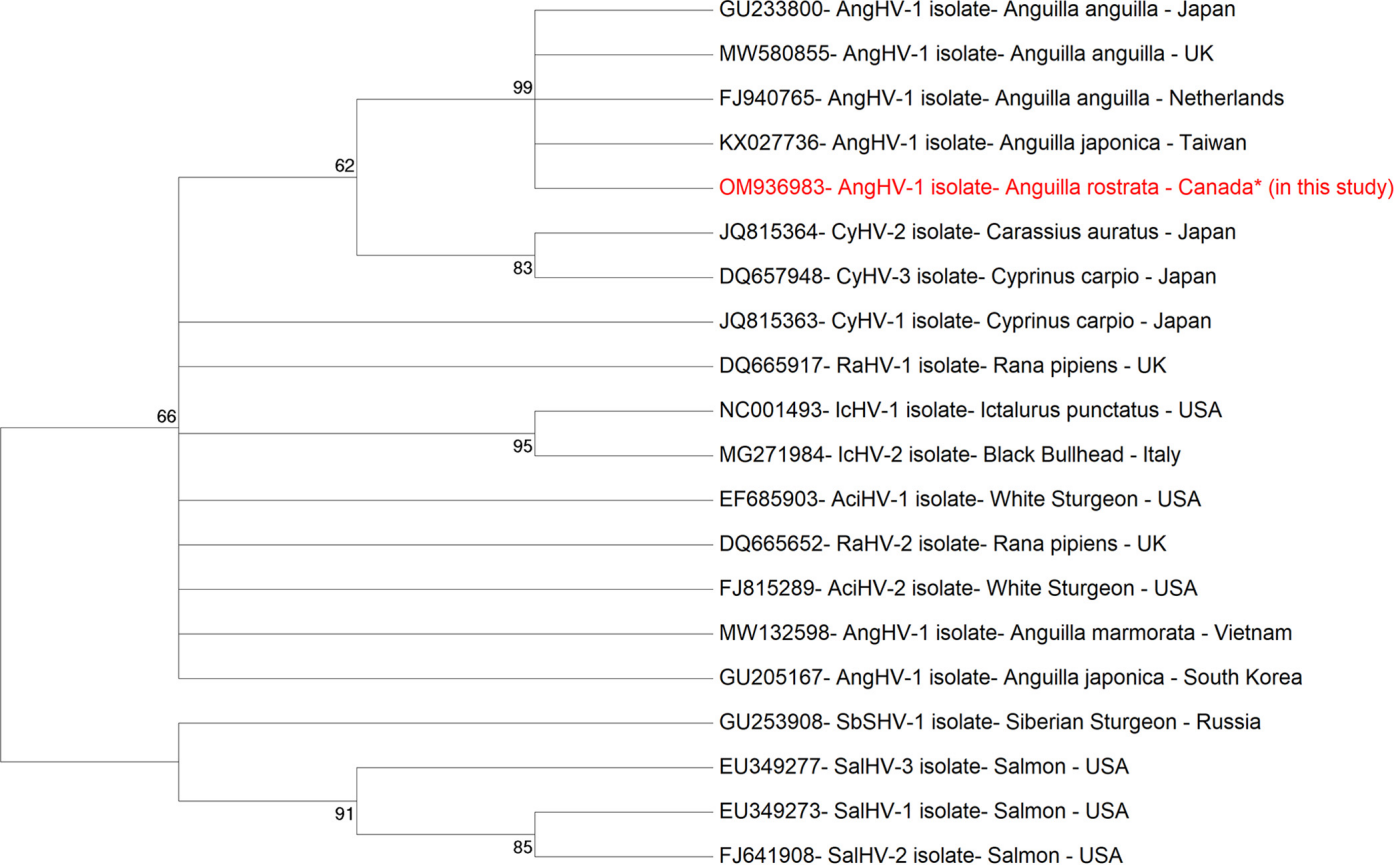
Unrooted maximum likelihood (ML)-based tree of the polymerase gene DNA sequences of AngHV-1 isolated from imported *Anguilla rostrata* from Canada and from records retrieved from GenBank. AciHV, acipenserid herpesvirus; AngHV, anguillid herpesvirus; CyHV, cyprinid herpesvirus; IcHV, ictalurid herpesvirus; RaHV, ranid herpesvirus; SalHV, salmonid herpesvirus; SbSHV, Siberian sturgeon herpesvirus. The GenBank accession number is provided for each sequence. The phylogenetic tree was constructed using MEGA7 software with the Kimura 2-parameter model, with the highest log likelihood. The bootstrap consensus tree inferred from 1,000 replicates with values above 50% is taken to represent the evolutionary history of the taxa analyzed. A discrete gamma distribution was used to model the evolutionary rate differences among sites (2 categories [*+G*, parameter = 10.8325]). The analysis involved 20 nucleotide sequences. The codon positions included were 1, 2, 3, and noncoding. All positions containing gaps and missing data were eliminated. There were a total of 353 positions in the final data set. The scale bar represents the number of substitutions per nucleotide site.

The sequence analysis report displaying the AngHV-1 virus isolated from *A. rostrata* indicated a complete genome length of 249,121 bp, including a pair of 11-kb terminal direct repeats and 133 protein-coding open reading frames (ORFs). The gDNA sequence is 99.7% and 99.4% identical to the previously reported sequences of AngHV-1 isolated from *A. japonica* from Taiwan (GenBank accession number KX027736) and from *A. anguilla* from the Netherlands (FJ940765), respectively. Further, phylogenetic analysis of the gene encoding DNA polymerase showed that the isolated virus was identical to sequences of AngHV-1 from other eel species (Fig. 1). However, a BLASTN search revealed a difference matrix of 0.01% to 3.98% for some ORFs encoding different viral proteins compared with the reported sequences (Table 1). Thus, it can be inferred that AngHV-1 has low genetic diversity among the different strains with respect to the host eel species or the geography of the isolation source.

**TABLE 1:** Results of a BLASTN search comparing the identity percentage of the AngHV-1 sequence in the present article with reference sequences

Protein-coding ORF	% identity of AngHV-1 isolated from *A. rostrata*^*a*^ with that isolated from:	
	*A. japonica* ^*b*^	*A. anguilla* ^*c*^
DNA packaging terminase subunit 1	100	100
ORF83	99.87	99.5
ORF18	100	100
ORF131	99.93	99.26
ORF34	99.39	99.79
DNA polymerase catalytic subunit	99.79	100
Allo56	100	100
ORF40	100	99.46
Allo37	99.9	99.8
ORF106	100	100
Membrane protein ORF67	100	99.83
ORF107	100	99.9
ORF86	99.98	100
ORF134	97.34	97.58
Major capsid protein	99.97	99.97
ORF1	99.45	96.02
ORF1	99.45	96.02
ORF45	100	99.97
Membrane protein ORF125	99.94	100
ORF30	100	99.97
ORF110	100	99.97
ORF120	99.97	100
Membrane protein ORF108	99.77	98.58
ORF127	99.93	99.93
ORF91	100	99.96
ORF48	100	99.88
ORF74	100	100
Ribonucleotide reductase subunit 1	100	100
ORF19	99.1	99.89
ORF44	100	100
Helicase-primase subunit	100	100
Membrane protein ORF109	100	100
Allo54	99.96	100
ORF23	100	99.96
Membrane protein ORF65	99.91	99.95
ORF31	100	100
Capsid maturation protease	99.26	100
Membrane protein ORF80	99.86	99.91
ORF87	99.95	100
ORF130	99.9	99.7
ORF111	99.95	99.95
ORF92	100	99.94
ORF113	99.43	99.94
ORF6A	98.84	98.78
ORF6A	98.84	98.78
ORF39	100	100
ORF20	100	100
Allo64	100	100
ORF88	99.94	99.94
Helicase-primase helicase subunit	100	100
ORF89	99.94	99.94
ORF28	100	100
ORF47	99.94	100
ORF85	100	98.03
ORF97	100	100
ORF61	100	100
ORF81	99.93	99.93
ORF99	99.93	100
Membrane protein ORF71	99.54	98.15
Membrane protein ORF101	100	100
ORF121	100	99.92
Membrane protein ORF66	100	100
ORF38	100	100
Membrane protein ORF95	99.91	100
Capsid triplex subunit 1	100	100
Allo60	100	100
Capsid triplex subunit 2	99.82	100
Membrane protein ORF94	99.72	99.91
ORF54	98.88	99.72
Membrane protein ORF93	100	99.9
Ribonucleotide reductase subunit 2	100	100
Uracil-DNA glycosylase	100	100
ORF112	99.76	100
Thymidylate synthase	99.89	100
ORF3	100	100
ORF3	100	99.89
ORF35	100	100
ORF16	100	100
ORF76	100	99.88
Deoxyguanosine kinase 1	100	99.88
ORF14	99.88	100
Membrane protein ORF124	100	100
ORF68	100	99.64
ORF13	99.88	100
ORF53	99.75	98.89
Membrane protein ORF11	99.75	100
Membrane protein ORF12	100	100
Deoxyguanosine kinase 2	100	100
ORF24	100	100
ORF4	100	96.95
ORF4	100	96.95
Membrane protein ORF50	100	100
Membrane protein ORF64	99.86	100
ORF58	100	99.86
Membrane protein ORF51	100	100
ORF62	100	100
ORF92B	100	100
ORF63	99.86	100
ORF17	100	100
Membrane protein ORF49	100	100
Dihydrofolate reductase	99.85	100
ORF105	100	100
Thymidylate kinase	100	100
ORF103	100	100
Membrane protein ORF8	100	100
ORF126	100	96.7
ORF46	100	100
Nucleoside diphosphate kinase	100	100
Membrane protein ORF102	100	99.67
ORF118	99.83	100
ORF117	100	100
Guanosine triphosphatase	99.65	100
ORF59	100	100
ORF41	100	100
ORF122	98.75	98.57
ORF114	100	100
ORF32	100	100
ORF60	99.81	100
ORF84	100	100
Deoxyuridine triphosphatase	100	100
Deoxyuridine triphosphatase	100	99.8
ORF56	100	100
Interleukin-10	100	99.8
ORF26	100	100
ORF43	100	100
ORF27	100	100
ORF33	100	100
Membrane protein ORF92A	100	100
Membrane protein ORF78	100	100
ORF69	100	100
ORF73	100	99.77
ORF70	100	100
ORF115	100	100

a AngHV-1 isolated from Anguilla rostrata from Canada; GenBank accession number OM936983.

b AngHV-1 isolated from *A. japonica* from Taiwan; GenBank accession number KX027736.

c AngHV-1 isolated from *A. anguilla* from the Netherlands; GenBank accession number FJ940765.

### Data availability.

The whole-genome sequence of AngHV-1 isolated from *A. rostrata* is available at NCBI under GenBank accession number OM936983. The raw data reads for the AngHV-1 genome sequence are available at NCBI under SRA accession number PRJNA883046. Images of diseased eels, AngHV-1 isolated in EK-1 cells, and transmission electron micrographs of AngHV-1 are available from Figshare (https://doi.org/10.6084/m9.figshare.20501703).
